# Aflatoxigenic *Aspergillus* Modulates Aflatoxin-B1 Levels through an Antioxidative Mechanism

**DOI:** 10.3390/jof9060690

**Published:** 2023-06-20

**Authors:** Bwalya Katati, Stan Kovacs, Henry Njapau, Paul W. Kachapulula, Bas J. Zwaan, Anne D. van Diepeningen, Sijmen E. Schoustra

**Affiliations:** 1 Laboratory of Genetics, Wageningen University and Research, 6708 PB Wageningen, The Netherlands; stan.kovacs@wur.nl (S.K.); bas.zwaan@wur.nl (B.J.Z.); sijmen.schoustra@wur.nl (S.E.S.); 2 Mycotoxicology Laboratory, National Institute for Scientific and Industrial Research, Lusaka 310158, Zambia; hnjapau@hotmail.com; 3 School of Agricultural Sciences, University of Zambia, Lusaka 10101, Zambia; paul.kachapulula@unza.zm; 4 Biointeractions and Plant Health, Wageningen University and Research, 6708 PB Wageningen, The Netherlands; anne.vandiepeningen@wur.nl

**Keywords:** aflatoxin-B1, antioxidant, atoxigenic *Aspergillus*, toxigenic *Aspergillus*

## Abstract

Aflatoxins (AFs) are considered to play important functions in species of *Aspergillus* section *Flavi* including an antioxidative role, as a deterrent against fungivorous insects, and in antibiosis. Atoxigenic *Flavi* are known to degrade AF-B1 (B1). To better understand the purpose of AF degradation, we investigated the degradation of B1 and AF-G1 (G1) in an antioxidative role in *Flavi*. Atoxigenic and toxigenic *Flavi* were treated with artificial B1 and G1 with or without the antioxidant selenium (Se), which is expected to affect levels of AF. After incubations, AF levels were measured by HPLC. To estimate which population would likely be favoured between toxigenic and atoxigenic *Flavi* under Se, we investigated the fitness, by spore count, of the *Flavi* as a result of exposure to 0, 0.40, and 0.86 µg/g Se in 3%-sucrose cornmeal agar (3gCMA). Results showed that levels B1 in medium without Se were reduced in all isolates, while G1 did not significantly change. When the medium was treated with Se, toxigenic *Flavi* significantly digested less B1, while levels of G1 significantly increased. Se did not affect the digestion of B1 in atoxigenic *Flavi*, and also did not alter levels of G1. Furthermore, atoxigenic strains were significantly fitter than toxigenic strains at Se 0.86 µg/g 3gCMA. Findings show that while atoxigenic *Flavi* degraded B1, toxigenic *Flavi* modulated its levels through an antioxidative mechanism to levels less than they produced. Furthermore, B1 was preferred in the antioxidative role compared to G1 in the toxigenic isolates. The higher fitness of atoxigenic over toxigenic counterparts at a plant non-lethal dose of 0.86 µg/g would be a useful attribute for integration in the broader biocontrol prospects of toxigenic *Flavi*.

## 1. Introduction

**Figure 1:**
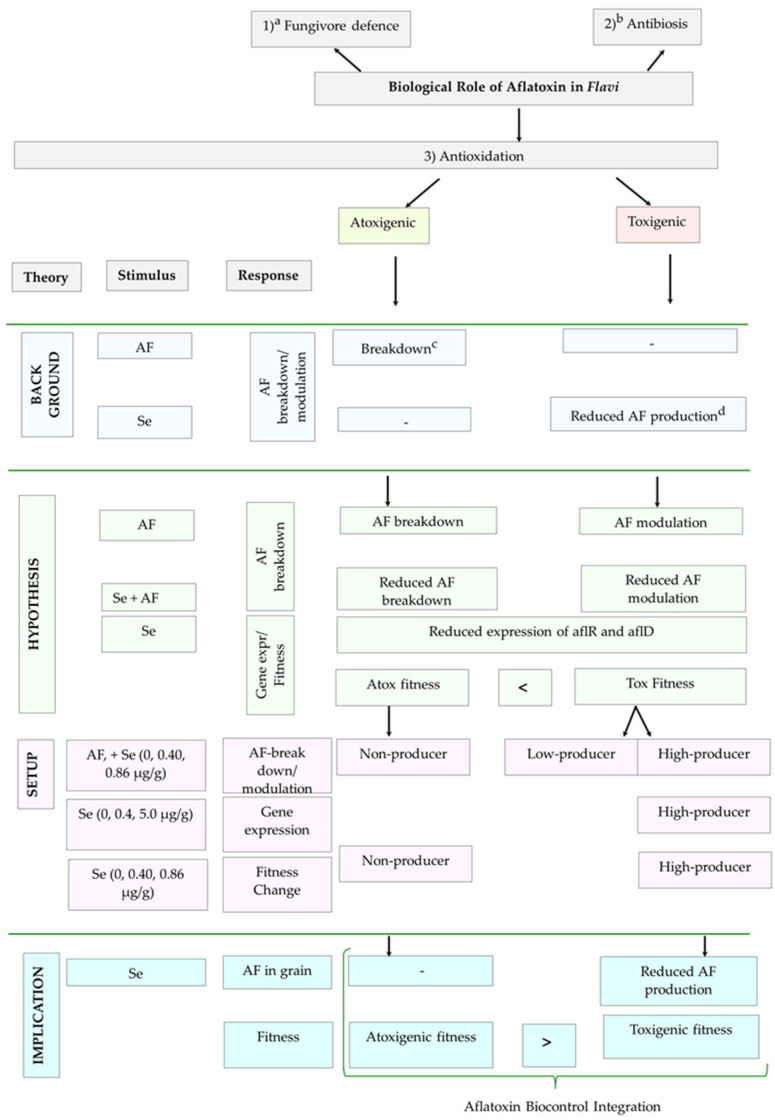
Schematic representation of the investigation of the role of aflatoxin (AF) degradation in an antioxidative mechanism. The scheme includes the background of what is known/presumed on breakdown of AF by atoxigenic *Flavi*, and reduction in AF production under increased antioxidant Se (selenium) in environment; hypothesis; experimental setup; implication on control of AF. ^a^ [17,18]; ^b^ [19]; ^c^ [33]; and ^d^ [20,21,22,23,24].

Aflatoxins (AFs) are a group of secondary metabolites produced by *Aspergillus* section *Flavi* (*Flavi*, for short) members such as *A. parasiticus* and *A. flavus*. The group of fungi infects important cereals such as maize and groundnuts [1,2,3,4,5,6], resulting in crop AF contamination. Of the known AFs produced by *Flavi*, AF-B1 (B1) is a highly mutagenic compound and is the most carcinogenic [7,8,9]. Among the known negative effects of B1 in both livestock and humans include growth impairment, reproductive system dysfunction, and immunosuppression [10,11,12]. In addition to this, studies have demonstrated the nephrotoxicity and acute hepatotoxicity of B1 on living tissue [13,14], including an effect on renal dysfunction among the negative effects on organs [15]. The oxidised form of B1 (AFB1-8, 9-epoxide) has been linked to cell injury due to its DNA mutagenicity [16]. The production of AF by *Flavi* has been demonstrated to be linked to useful biological functions, including the competitive inhibition of other microbes such as fungivores [17,18], antibiosis against bacteria [19], and oxidative stress alleviation [20,21,22,23,24] (Figure 1).

The link of AF to oxidative stress alleviation suggests that AF may be responsible for the scavenging of reactive oxygen species (ROS) [25], which results in the subsequent degradation of the AF molecule in reaction with the ROS. In this regard, it has been suggested that an increase in the antioxidative capacity of a plant could alleviate the increase in AF production by aflatoxigenic species such as *A. flavus* [22]. Hence, it would be estimated that introducing an antioxidant in the growth environment of the fungus (the crop) would reduce ROS, thereby reducing the need for the fungus to produce AF. A number of antioxidant elements are known, which include copper, manganese, selenium, and zinc. Selenium (Se) is important to human health [26] as well as plays an anti-carcinogenic role [27]. The element is also known to play a defensive role against AF in animals [28,29,30]. In the right amounts, Se can enhance plant growth [31].

Se can be introduced into a plant by biofortification through conventional breeding, genetic modification, mineral fertilisation of the soil, or through foliar application. In crop protection, the biofortification of plants with such an element would play the role of indirect control of AF accumulation in important cereals such as maize and wheat while improving the crop’s micronutrient status. However, it should also be noted that Se can be toxic in elevated levels to the plant itself [31,32] other than inhibiting fungal sporulation at high levels.

Atoxigenic *Flavi* strains have been demonstrated to degrade B1 [33]. With respect to this, colonisation of a crop by atoxigenic *Flavi* has been demonstrated to be able to reduce AF contamination in crops such as maize, cotton, and groundnuts [34,35,36]. Although the degradation of AF by atoxigenic *A. flavus* has been demonstrated [33], the reason behind the phenomenon is still unclear. For example, considering that atoxigenic *Flavi* do not produce AF, the question arises whether or not the degradation of AF could have a link to antioxidative alleviation in the non-aflatoxin producing *Flavi*, and if the mechanism would be the same as that for aflatoxin producers.

Some studies have demonstrated that the mechanism of inhibition of AF production involves the downregulation of the regulatory gene *aflS*, reducing the abundance of the ratio of *aflS* to *aflR* [37]. This would imply that there is an actual reduction in the amount of AF produced by the *Flavi* due to reduced messenger RNA from structural genes such as *aflD*, and not necessarily the reduction in AF through its degradation. The *aflD* is a structural gene responsible for the early conversion of AF precursor molecules in the aflatoxin biosynthetic pathway [38,39] and is regulated by the regulatory genes *aflR* [40,41,42,43] and *aflS*. To enhance the understanding of the potential role of AF production and its utilisation in *Flavi*, two related questions need to be answered: (1) is AF degraded by atoxigenic *Flavi* as part of oxidative stress alleviation, and (2) is AF also degraded by aflatoxin-producing *Flavi* (toxigenic) as part of the mechanism of oxidative stress alleviation?

Introducing a natural antioxidant such as Se to the environment of toxigenic and atoxigenic members of section *Flavi* and observing the associated levels of aflatoxin produced in its environment might provide some insights into the potential role aflatoxin plays as an antioxidant in both toxigenic and atoxigenic *Flavi*. It would also help to understand in which direction the population dynamics between toxigenic and atoxigenic *Flavi* would likely shift in a Se-fortified environment. It is known that fungi respond to abiotic stimuli, and that the exact nature of the response will depend on the fungal strain and abiotic condition. With respect to an antioxidative environment, if aflatoxin were an antioxidant for both toxigenic and atoxigenic *Flavi*, then one would expect that toxigenic *Flavi* would exhibit a higher Darwinian fitness (e.g., produce relatively more spores) in an environment with antioxidants such as Se, compared to atoxigenic *Flavi*. It would, however, be desirable that atoxigenic strains are fitter in a Se-fortified environment compared to toxigenic counterparts (Figure 1), such that population dynamics are not in favour of the toxigenic over atoxigenic strains.

The aim of this investigation was to explore the involvement of AF as an antioxidant in the growth environment for toxigenic and atoxigenic *Flavi*. The specific objectives of this study were to (1) determine whether AF is broken down by both atoxigenic and toxigenic *Flavi* in the role of antioxidation and (2) to establish differences in fitness levels between atoxigenic and toxigenic isolates as a result of antioxidant (Se) fortification. We hypothesise that both toxigenic and atoxigenic *Flavi* utilise AF in an antioxidative role. We also hypothesise that toxigenic strains are fitter when treated with antioxidant Se compared to atoxigenic counterparts. We base our second hypothesis on grounds that toxigenic isolates expend more energy producing AF, presumably in an antioxidative role, compared to the atoxigenic strains. This would imply that the introduction of a substitute antioxidant (Se) relieves more energy for the aflatoxin-producing fungus to utilise for other purposes, including sporulation (fitness).

Understanding the role of AF in both toxigenic and atoxigenic *Flavi* from an antioxidative role will better guide efforts aimed at improving the micronutrient status of maize without the risk of promoting toxigenic isolates over atoxigenic counterparts if integrated into the broader biocontrol programme. In addition, such knowledge will help to better understand how antioxidants would help in preventing spikes of AF in maize.

## 2. Materials and Methods

The *Aspergillus* section *Flavi* isolates used were collected as live wild-type laboratory cultures stored in glycerol (−70 °C). They had previously been isolated from maize and soil (data unpublished). Isolates were regenerated on Malt Extract Agar medium (MEA) for 7 days (25 °C, dark). Spores from regenerated cultures were suspended in 0.8% sterile saline solution as stock culture solution. The stocks were diluted with sterile MilliQ water to 1 × 10^4^ spores/mL prior to inoculations. Chromatographic analyses for aflatoxin (AF) were performed by HPLC (Agilent Infinity II 1260 Series, Agilent Technologies, Santa Clara, CA, USA). Technical standards for AF-G2, -G1, -B2, and -B1 were obtained from Merck (Merck Life Science NV, Amsterdam, The Netherlands).

### 2.1. Determination of Antioxidative Degradation of Aflatoxin by Toxigenic and Atoxigenic Flavi

To determine the antioxidative role of aflatoxin (AF) in its degradation by *Flavi*, we specifically used an antioxidant (selenium) in lieu of previously used oxidative compound such as H_2_O_2_ [22,24]. This would enable the direct determination of the role of AF in antioxidation, by using a substitute antioxidative compound (Se).

#### 2.1.1. Assignment of Atoxigenic and Toxigenic Isolates

From the regenerated lab culture isolates, we selected 12 wild-type laboratory cultures preliminary screened for aflatoxin production ability based on qualitative visualisation of their aflatoxin (AF) bands by Thin Layer Chromatography (TLC) [44]. The first four isolates were initially assigned as non-producers (atoxigenic) based on absence of AF bands on TLC plates. Another four isolates were initially assigned as high producers based on very high intensity of AF bands on TLC plates. Then, four isolates were assigned as low producers based on less intense bands of Aflatoxins by TLC compared to the high producers. All 12 isolates were then verified by HPLC for levels of AF produced. To determine this, a 100 µL spore suspension (1 × 10^4^ spores/mL) per isolate was inoculated into test tube containing 2 mL autoclave sterilised Yeast Extract Sucrose (YES) broth (200 g/L sucrose; 20 g/L Yeast Extract autolysate, pH 6.0) [45]. Tubes were incubated at 31 °C (7 days, dark). Aerobic liquid fermentation was then carried out with shaking at 150 rpm in a temperature-controlled incubator (Innova 4330, New Brunswick Scientific, Enfield, CT, USA). AF was extracted by dichloromethane partitioning, adding 2 mL dichloromethane to the YES broth. Tubes were then vortexed for 45 s on a vortex unit (Genius model VG 3 S000, IKA, Staufen, Germany). Tubes were then centrifuged at 25,000× *g* (microcentrifuge model 5424, Eppendorf, Hamburg, Germany) for 5 min. The supernatant (aqueous phase) was pipetted off and discarded. Next, 500 µL of the dichloromethane phase (lower phase) was transferred into a 4 mL screw neck vial (45 × 14.7 mm, VWR, cat No. 548-0051). The dichloromethane fraction was then evaporated to dryness and then dried material reconstituted in 250 µL 1:1 methanol/water (*v*/*v*). For AF producers, the reconstituted material was diluted 10-fold into an HPLC vial, then 10 µL injected into HPLC. For non-producers, reconstituted material was diluted to 500 µL total volume into HPLC vial, then 20 µL was injected. Reversed-phase separation of injected analyte was achieved with a Cortecs C18 column of 3.5 i.d × 100 mm × 2.7 µm particle size (Waters, Milford, MA, USA) fitted with a guard column. The mobile phase constituted 0.1%-acetic acid/methanol/acetonitrile 70:20:10 (*v*/*v*) at a flow rate of 0.45 mL/min. The column oven was set at 35 °C and the eluted AFs were detected on fluorescence detector (λexcit = 365 nm; λemit = 440 nm). Quantification of AFs was achieved with Chemstation software. The isolates were hence assigned as high (>300 ng AF-B1+G1/mL YES medium), low (LOQ—299 ng AF-B1+G1/mL YES), and non-producers (Table 1). Limit of quantitation (LOQ) in AF extracts was assigned as 2.5 ng/mL. All values below LOQ were converted to 0 as unquantifiable noise.

#### 2.1.2. Aflatoxin (AF) Degradation Assay

Artificial AF was first prepared from one aflatoxin-producing isolate, “EKZ10A.” For this, a 100 µL spore suspension (1 × 10^4^ spores/mL) of the isolate EKZ10A was inoculated into test tube containing 2 mL autoclave sterilised YES broth [45] (pH 6.0). Fifty tubes were incubated at 31 °C (7 days, dark) for the liquid fermentation with shaking at 150 rpm in a refrigerated incubator (Innova 4330, New Brunswick Scientific, Enfield, CT, USA). AF was similarly extracted by dichloromethane partitioning as described above, collecting the centrifuged dichloromethane (CH_2_Cl_2_) extracts from each tube into one pooled sterile 50 mL tube to ensure homogeneity of AF concentration. Next, 1000 µL of AF extract in CH_2_Cl_2_ was transferred per 10 mL glass test tube (×104 tubes) into ×96 test samples, ×4 positive controls, and ×4 negative controls. The CH_2_Cl_2_ was vapourised under fume hood to dryness and reconstituted with 300 µL absolute ethanol to re-sterilise artificial AF. Tubes were then placed in laminar flow hood to vaporise the ethanol. Next, 2 mL of sterilised YES agar medium was dispensed warm at 50 °C per tube, then vortexed for 20 s to redissolve the AF extract prior to isolate inoculation. The YES agar constituted 0.2% agar to generate a light paste. One set of YES agar had been fortified with anhydrous sodium selenite (analytical reagent) at intermediate concentration of 0.40 µg/g below a plant lethal dose of 5 mM (equivalent to 0.86 µg/g). In this experiment, the selenite salt was the antioxidant. The YES tubes were set up for the 12 isolates, with each isolate receiving all four of the following treatment permutations: (i) isolate only (“Spp”), (ii) isolate with AF (“Spp+Afl”), (iii) isolate with antioxidant (“Spp+AOx”), and (iv) isolate with AF and antioxidant (“Spp+Afl+AOx”). Each isolate was duplicated (total of 96 test samples). Positive controls to determine base AF levels in tubes were AF extract without isolate (“Afl”) as well as AF extract with antioxidant (“Afl+AOx), in duplicate. The spiking concentration of AF in the tubes was determined based on concentration of the positive controls. This was done post-incubation and determined by HPLC as described in the above section. Hence, the average spiked concentration of the AF in the incubated tubes was G2 = 150 ± 35 ng/mL; G1 = 3244 ± 786 ng/mL; B2 = 86 ± 6 ng/mL; and B1 = 2114 ± 68 ng/mL. Negative controls were YES agar as well as YES agar with antioxidant, in duplicate. Each tube with the YES agar was inoculated with 50 µL of 1 × 10^4^ spores/mL isolate suspension for each of the 12 test isolates. The positive controls were inoculated with 50 µL sterile Milli-Q H_2_O in place of isolate inoculation. All tubes were incubated at 31 °C (7 days, dark). The liquid fermentation was carried out in an incubator (Innova 4330, New Brunswick Scientific, Enfield, CT, USA) with mild shaking at 100 rpm. After the incubation, AF was extracted by dichloromethane partitioning, as described in the above section. No biomass growth ensued in the positive controls for AF, confirming integrity in sterility of the artificial AF extract medium during preparation. AF was then quantified by HPLC with autosampler as described above.

1. Gene Expression (*AflR* and *AflD*) Response to Antioxidant (Se)

To determine the possible correlation response of gene expression to antioxidant treatment, three randomly selected high-aflatoxin-producing isolates (EKZ10B, ELG33C, and MLV14F) were tested for their gene expression correlation response. Unfortunately, isolate EKZ10B fell out of the experiment. Target genome loci were *aflR* and *aflD* (Table 2), which are part of the aflatoxin biosynthetic pathway genes. The β-tubulin was used as the endogenous control to normalise the qPCR cycle threshold (Ct) values.

2. Generation of RNA

For generation of the genomic RNA with response to antioxidant, the spore suspensions from isolates ELG33C and MLV14F were plated on cellophane discs immersed in YES agar fortified with Se. YES was used as it induces AF production. The antioxidant was introduced as mineral Na_2_SeO_3_ at concentrations in µg/g YES of 0 (RNA calibrator), 0.40 (intermediate Se concentration below a plant lethal dose of about 5 mM or 0.86 µg/g), and 5.0 (elevated concentration). Treatments were incubated in triplicate at 31 °C (12 days, dark).

3. Isolation and Quantification of RNA

Mycelia were aseptically harvested from top of cellophane disc into an RNA-/DNA-ase free (sterile) 1.5 mL screw cap tube containing four sterile 3 mm glass beads. Each tube, upon harvest of mycelia, was immediately placed into liquid N2. The frozen tubes were then clumped on a sideways beater (model MM400, Retsch, Haan, Germany), and beating was performed at 30 beats/second for 30 s for the cell disruption. Immediately, 800 µL TRI lysis solution (RNA lysis buffer) was added per tube and inverted three times to suspend all material in the tube. For the rest of the isolation steps, the Zymo Research Kit (catalogue No. R2073, Zymo Research, Irvine, CA, USA) was used, following the RNA extraction protocol for “Tough-to-Lyse Samples.” The vortex was performed at max speed of 10 (Genius model VG 3 S000, IKA, Staufen, Germany) for 15 s. Centrifuging was performed at 16,000× *g* (centrifuge model 5424, Eppendorf, Hamburg, Germany) at room temperature. Extracted RNA was eluted from column with 100 µL sterile DNase/RNase-free Milli-Q water. The RNA was stored at −80 °C for long-term storage or −20 °C for short-term storage for downstream analysis.

Purified total RNA concentration was measured using the Qubit fluorometer (Invitrogen, Thermo Fisher Scientific, Waltham, MA, USA) using the RNA BR Assay Kit. The RNA quality was analysed directly on the nanodrop (model 2000, Thermo Fisher Scientific, Wilmington, DE, USA) using 1.0 µL extracted RNA per sample (A260:A280 ratios: ELG33C = 1.91 ± 0.07; MLV14F = 1.67 ± 0.00). Furthermore, the RNA integrity was checked with 1% gel electrophoresis, mixing 8 µL RNA with 2 µL loading dye. Two clear bands were obtained, indicating the ribosomal material had not degraded.

4. cDNA Synthesis (Reverse-transcriptase PCR)

The complementary DNA (cDNA) was synthesised using the SensiFast^®^ cDNA synthesis kit (Bioline, Luckenwalde, Germany) in a 20 µL reaction mixture as follows: 15 µL (~500 ng ELG33C; ~200 ng MLV14F) of mRNA template was added to a 0.5 µL PCR tube. Next, 4 µL 5X TransAmp buffer was added per 0.5 PCR tube, followed by addition of 1 µL enzyme Reverse Transcriptase. Contents were gently mixed by up–down pipetting. The cDNA was synthesised from the mRNA in a thermal cycler (model T100™, Biorad Laboratories Inc., Hercules, CA, USA) under the following conditions: primer annealing, 25 °C × 10 min; reverse transcription, 42 °C × 15 min; optional step (in case of highly structured RNA), 48 °C × 15 min; and inactivation, 85 °C × 5 min. The synthesised cDNA concentration was then extrapolated from the RNA concentration prior to the cDNA synthesis. The cDNA concentration was then diluted to 3.5 ng/µL.

5. Quantitative PCR (Real-time PCR)

Real-time PCR (qPCR) was carried out in a Real-Time Thermal Cycler (model CFX96™, Biorad Laboratories Inc., Hercules, CA, USA) using the iQ SYBR Green Supermix kit. For test samples, the qPCR was carried out in triplicate in total reaction volume of 10 µL per sample in MicroAmp optical 96-well reaction plates, sealed with optical adhesive covers (Applied Biosystems, Life Technologies, Waltham, MA, USA). Each reaction was performed using 5 µL of 2X iQ SYBR Green supermix, 2 µL of 2 µM reverse primer (400 nM), 2 µL of 2 uM forward primer (400 nM), and 1 µL of cDNA template of 3.5 ng/µL. For primer efficiency check, DNA from isolate EKA03C, already verified positive for *aflR* and *aflD*, was used, which was serial diluted to 3.50 ng/µL, 1.75 ng/µL, and 0.875 ng/µL. Negative control consisted sterile Milli-Q water in place of DNA. The thermal cycling conditions were executed with an initial denaturing step of 95 °C × 2 min followed by 40 cycles of 95 °C × 15 s (denature), 55 °C × 20 s (anneal), and 72 °C × 20 s (elongate). Melting curve analysis of the PCR product was performed by ramp heating from 50 °C to 95 °C in steps of 0.5 °C per increment, continuously measuring the fluorescence. Each ramp per increment was held for 20 s, while Ct values were acquired from instrument. The housekeeping gene β-Tubulin was used as the endogenous expression control against which normalisation of Ct values for *aflR* and *aflD* was performed.

6. Corresponding Aflatoxin Production with Gene Expression

Pieces of YES agar under the cellophane on which RNA generation had been performed were collected into a pre-weighed test tube. The agar was crushed to paste with clean rod and was re-weighed in the test tube. A total of 5 clean glass beads (Ø 3 mm) were added per tube, and 600 µL of 0.05% Triton-X was added per tube. Contents were vortexed for 10 s continuously at max speed of 10 on a vortex unit (Genius model VG 3 S000, IKA, Staufen, Germany). Next, 3 mL dichloromethane was added per gram agar, and the AFs were extracted by dichloromethane partitioning, as described above.

### 2.2. Fitness Response of Atoxigenic and Toxigenic Isolates under Antioxidant (Se)

#### 2.2.1. Isolates Collection and Preparation of Inoculation Medium

From the retrieved and regenerated isolates of maize and soil, we selected five toxigenic and five atoxigenic isolates on the basis of their high sporulation ability without Se treatment. Spore count was used as the proxy for isolate fitness. CMA amended with 3% *w/v* sucrose (3gCMA) was used for isolates fitness assays. The 3gCMA was prepared by adding 17 g cornmeal agar per litre of demi water, according to manufacturer specifications. Next, 3% *w/v* sucrose was added to the medium, configuring the medium to more or less mimic the kernel, including its sucrose-rich endosperm. Three levels of Se concentration in the 3gCMA were prepared by fortifying the medium with Se at 0 µg/g, 0.40 µg/g (intermediate Se concentration below a plant lethal dose of about 5 mM or 0.86 µg/g), and 0.86 µg/g maximum (equivalent maximum non-homeostasis-toxic mineral Se concentration in maize). The medium was then amended to 50 mg/L chloramphenicol and then boiled to dissolve the contents. Medium was then dispensed warm and viscous (50 °C) to 50 mL glass bottles at 3 mL/bottle. Bottles were autoclaved at 120 °C × 15 min.

#### 2.2.2. Fungal Fitness Assay under Antioxidant (Se) Treatment

Isolate spore suspensions, adjusted to 1 × 10^4^ spore/mL, were inoculated in triplicate at 20 µL per sterile 3gCMA bottle with or without Se. The spore suspension was spread on the agar using 3 mm glass beads. Beads were discarded, and bottles were incubated at 30 °C (7 days, dark). Spores were then harvested from the medium with 0.05% Triton-X (surfactant). For the harvest, 3 mL surfactant was added per bottle, and contents were placed on sideways shaker (GFL model 3018, Society for Laboratory Technology, Burgwedel, Germany) and agitated at 200 rpm per minute for 2 min. Next, 1000 µL spore suspension was transferred by pipettor while swirling to a 1.5 mL microcentrifuge tube. Spore concentration was analysed using an automated cell counter (Casy™ TT, Omni Life Sciences, Bremen, Germany). Briefly, 10 µL of the collected spore suspension was added to 10 mL of Casy Ton dilution solution, and contents were gently inverted 10 times, avoiding build-up of bubbles. Immediately, the count was performed on the unit. If spore count was below 200 counts detected, 100 µL spore suspension was used in the dilution in place of 10 µL.

### 2.3. Data Analysis

To determine the degradation of AF with or without antioxidant treatment, the pairwise Wilcoxon rank sum exact test was used on the treatments. Due to the relatively low yield of the AF variants B2 (86 ng/mL) and G2 (150 ng/mL) in the artificial AF, assays were only performed on B1 (2114 ng/mL) and G1 (3244 ng/mL) in order to generate statistically viable results. G2 and B2 are the more oxidised forms of AF compared to G1 and B1, which are the more reactive forms of aflatoxin for such a redox assay and are the more important in terms of carcinogenicity. Relative gene expression was calculated using the 2^−ΔΔCt^ method, as described by [48]. For gene expression, we considered either the upregulation or downregulation of *aflR* and *aflD* by relative number of times the expression increased or reduced in relation to calibrator (0 Se treatment). The simultaneous upregulation or downregulation of *aflD* and *aflR* was tested by Spearman rank correlation rho.

Fungal fitness was determined as spore concentration and analysed across treatments by Wilcoxon rank sum exact test, significant if *p* < 0.05. Mean spore counts between atoxigenic and toxigenic isolates were determined as geometric mean. Data computations were executed in software R [49] version 4.1.0. Visualisations were computed with aid of the R package ggplot2 [50].

## 3. Results

### 3.1. Determination of Antioxidative Degradation of Aflatoxin

**Figure 2:**
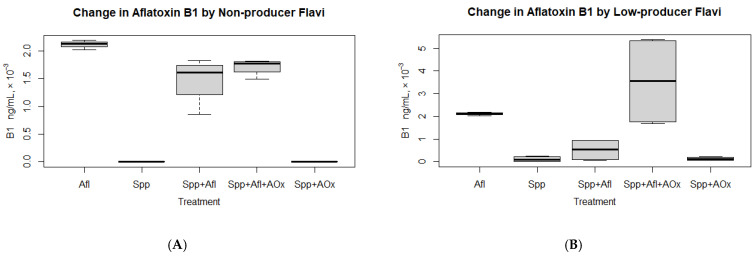

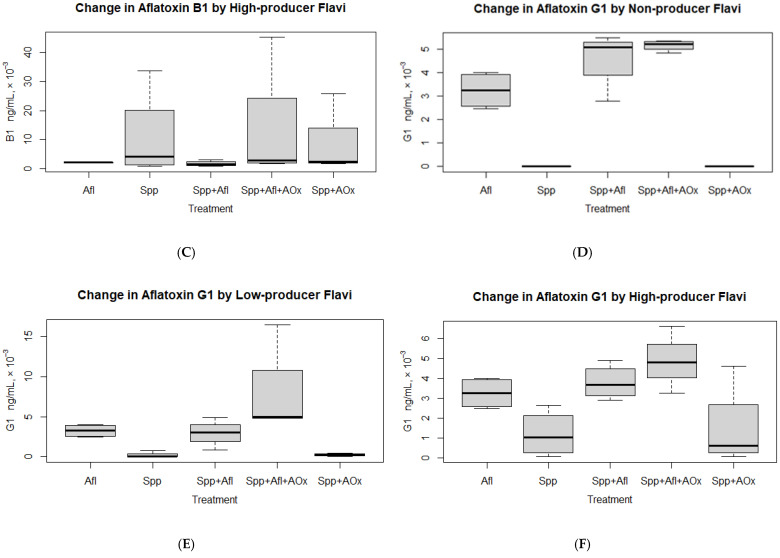
Boxplot showing change in quantity of AFs B1 (**A**–**C**) and G1 (**D**–**F**) exposed to toxigenic and atoxigenic *Flavi* with or without the antioxidant (Se). (**A**,**D**) is change in B1 and G1 in none-producers; (**B**,**E**) is change in B1 and G1 in low-producers; and (**C**,**F**) is change in B1 and G1 in high-producers.

Without the antioxidant, the results of the change in AF (B1 and G1) are shown across the variables “Afl,” “Spp” and “Spp+Afl” (Figure 2), summarised in Table 3. With the antioxidant, the change in B1 or G1 is shown across the variables “Spp+Afl+AOx” and “Spp+AOx” (Figure 2), summarised in Table 4. “Afl,” is the extrinsically introduced B1 or G1, while “Spp” is the toxigenic or atoxigenic *Flavi*. “AOx” is the antioxidant selenium (Se). 

#### 3.1.1. Degradation of Aflatoxin in Non-Aflatoxin Producers without Antioxidant Treatment

There was a significant reduction in added B1 (“Afl”) when exposed to the atoxigenic strains (“Spp+Afl”) (Figure 2A; Table 3), with all isolates digesting the toxin. The greatest reduction was observed with isolate 1MS7 (60%). There was, however, no significant reduction in G1 added. All isolates except 1MS7 did not reduce the added G1. Isolate 1MS7 reduced G1 by 14%.

#### 3.1.2. Change in Aflatoxin in Low-Aflatoxin Producers without Antioxidant Treatment

There was a significant reduction in B1 due to exposure of added AF (“Afl”) to low AF producer strains (“Spp+Afl”) (Figure 2B; Table 3). All isolates reduced the B1. There was no significant overall reduction or change in G1 levels compared to the added amount (“Afl”) due to exposure to low AF producer strains (“Spp+Afl”). Two isolates reduced G1, with ELV13C having the highest G1 reduction (73%). The two other isolates did not reduce G1. Furthermore, the maximally produced levels of G1 (“Spp” = 732 ng/mL) were exceeded when extrinsic AF (G1 “Afl” = 3243 ± 786 ng/mL) was introduced (G1 “Spp+Afl” = 4881 ng/mL).

#### 3.1.3. Change in Aflatoxin in High-Aflatoxin Producers without Antioxidant Treatment

There was no significant change in added B1 (“Afl”) due to exposure to high AF producer strains (“Spp+Afl”) (Table 3). However, the B1 maximum levels produced (“Spp+Afl”: 3169 ng/mL) were modulated to levels closer to the added amount (“Afl”: 2114 ± 68 ng/mL) compared to the maximum levels the species (“Spp”) would produce without extrinsic B1 (“Spp”: 33,749 ng/mL) (Figure 2C). (Modulate implies maintaining the level of the aflatoxin variant to within what the *Flavi* produces.)

With G1, the change was not significant between amount the species produced (“Spp”: 1181 ng/mL) and what it produced together with extrinsically introduced G1 (“Spp+Afl”: 3793 ng/mL) (Table 3). However, the total amount of G1 (“Spp+Afl” = 3793 ng/mL) simulated an augmentation of added G1 amount (“Afl” = 3243 ± 786 ng/mL) with the amount produced by the species (“Spp” = 1181 ng/mL) (Figure 2F).

Overall, the aflatoxin-producing isolates modulated B1 in their environment by reducing the extrinsically added amount or limiting the overall amount to levels added. This was, however, not the case with G1, which tended to increase overall (Table 3).

#### 3.1.4. Degradation of Aflatoxin in Non-Aflatoxin Producers with Antioxidant (Se) Treatment

The antioxidant did not lead to a significant change in extrinsic B1 or G1 reduction levels. (Wilcoxon rank sum exact test, “Spp+Afl” v “Spp+Afl+AOx” *p* > 0.05). As expected, treatment of the isolates with the antioxidant did not change the AF levels (remained < LOQ) as the isolates were non-producers.

#### 3.1.5. Change in Aflatoxin in Low-Aflatoxin Producers with Antioxidant (Se) Treatment

The antioxidant in the presence of extrinsic B1 led to an increase in the levels of B1 (“Spp+Afl” v “Spp+Afl+AOx”, Table 4; Figure 2B). When antioxidant was introduced to the species without B1 (“Spp+AOx”), the maximum levels of AF the species could produce did not exceed that produced by the species without treatment (“Spp”). With G1, there was no significant difference in levels with or without treatment with antioxidant as long as G1 had been introduced extrinsically, showing ineffectiveness in the overall breakdown of G1 by the “Spp” with or without antioxidant (Table 4, Figure 2E). There was an observed reduction in maximum levels of G1 produced when “Spp” had been treated with antioxidant (“Spp+AOx”), although the overall difference in G1 between “Spp” and “Spp+AOx” was not significant.

#### 3.1.6. Change in Aflatoxin in High-Aflatoxin Producers with Antioxidant (Se) Treatment

There was no effect of antioxidant in the presence of extrinsic B1 on overall levels of B1 (“Spp+Afl+AOx” v “Spp+Afl”). However, the maximum levels of B1 increased in the presence of antioxidant from 3169 to 45,168 ng/mL, showing a reduction in the modulation of the maximum levels of B1 in the presence of antioxidant.

When antioxidant was introduced to the species without extrinsic B1 (“Spp+AOx”), the maximum levels of AF the species could produce did not exceed that produced by the species without treatment (Table 4, Figure 2C). The overall difference in generated B1 between the treatment of species with antioxidant and non-treatment was, however, not significant. With G1, there was no significant difference in levels of G1 with or without treatment with antioxidant (“Spp” v “Spp+AOx”, *p* > 0.05) as long as G1 had been introduced extrinsically (Figure 2F). This showed that G1 was not effectively broken down by the “Spp” with or without antioxidant. However, there was an observed increase in maximum levels of the G1 produced in the presence of antioxidant (Table 4).

Considering that high producer strains did not significantly change levels of AF produced due to low antioxidant dose (Se = 0.40 µg/g), we investigated the gene expression patterns of structural gene *aflD* and regulatory gene *aflR* as well as AF response in two selected high AF producer strains (MLV14F and ELG33C).

##### Gene Expression in Aflatoxin Pathway Genes in High Producer Isolates Due to Antioxidant

**Figure 3:**
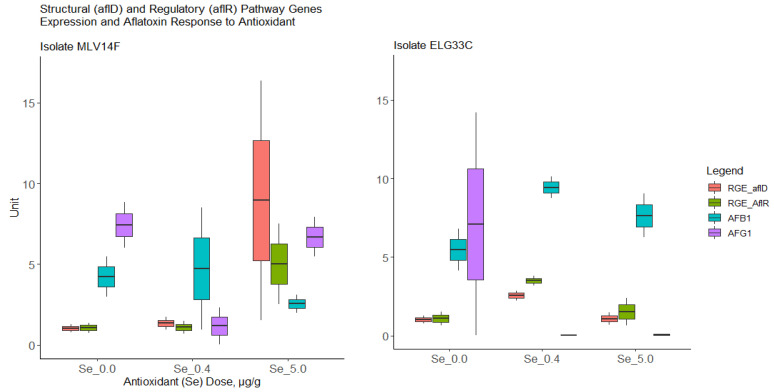
Relative gene expression change at different selenium (antioxidant, Se) treatment levels and change in B1 and G1 production in *Flavi* lab culture isolates. AFB1 = Aflatoxin-B1, µg/g; AFG1 = Aflatoxin G1, µg/g. The aflatoxin levels (µg/g) are concentrations in agar upon elapse of 12-day incubation. Abbreviations: RGE = relative gene expression; Se = selenium.

It was observed from the two randomly tested isolates that in both, the up-regulation of the regulatory gene *aflR* led to the up-regulation of structural gene *aflD* (which is involved in AF precursor molecule decoration) and vice versa (Figure 3; Spearman correlation: rho = 0.90; *p* value < 0.001).

However, the two isolates responded differently to the levels of the antioxidant introduced. Isolate ELG33C showed an upregulation of genes at Se dose 0.4 µg/g and a downregulation at a much higher (8-fold) antioxidant dose. However, the isolate MLV14F upregulated genes at a much higher Se level (8-fold) with no effect at a 0.4 µg/g Se dose. There was no clear correlation between gene expression in the two pathway genes and AF levels. However, it was exceptionally observed that B1 in isolate ELG33C, whose gene upregulation responded to lower Se levels, increased with the upregulation of *aflR* and *aflD* and reduced with the downregulation of the two genes.

### 3.2. Fitness of Isolates Due to Antioxidant Treatment

**Figure 4:**
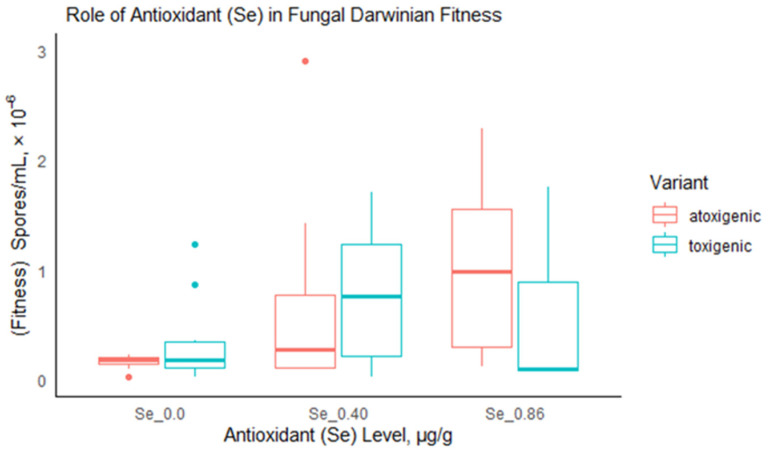
Boxplot of the change in *Flavi* fitness as a result of Se antioxidant treatment levels in µg/g 3% sucrose CMA. The difference in the fitness between toxigenic and atoxigenic *Flavi* at Se treatment level 0.86 µg/g was significant (*p* = 0.023).

Without antioxidant (Se) treatment, both atoxigenic and toxigenic fungi were of equal fitness (Wilcoxon rank sum exact test, *p* = 0.739). Similarly, there was no significant difference in fungal fitness at antioxidant treatment level 0.40 µg/g (*p* = 0.912). However, a difference in fitness was detected at the treatment level 0.86 µg/g (Figure 4, *p* = 0.023). The non-producer isolates were fitter than their aflatoxin-producing counterparts (geometric mean: atoxigenic = 0.655 × 10^6^ spores/mL and toxigenic = 0.209 × 10^6^ spores/mL). 

## 4. Discussion

### 4.1. Degradation of Aflatoxin Levels in Flavi through Antioxidative Mechanism

With the investigated strains, we demonstrate that toxigenic *Flavi,* in fact, modulate levels of B1 in their environment to within the levels they produce it. To do so, they partly degrade the B1 and also maintain the B1 to within a certain level. The modulation is evidenced by the reduction in the added amount of B1 to medium, to levels within what the species produced (“Afl” v “Spp+Afl” in Figure 2B,C). Furthermore, we demonstrate that this is carried out through an antioxidative mechanism, such that B1 plays an antioxidative role. The antioxidative role is evidenced by the increase in levels of AF in the medium in which both AF and selenium (Se) had simultaneously been added compared to AF alone without the addition of Se (“Spp+Afl” v “Spp+Afl+AOx” in Figure 2B,C). The mechanism in non-producers may, however, not be the same as for producers, as seen by the no effect of Se on the breakdown of extrinsically introduced AF (Figure 2A, Table 4). The observed increase in levels of B1 suggests that the antioxidative role of B1 as a free-radical scavenger was demoted by the presence of the antioxidant Se, with Se presumably taking the more active role in the wiping out of oxidative elements. Examples of such oxidative elements include reactive oxygen species (ROS), as previously described [20,22,24]. Our results also show that B1 is the preferred natural antioxidative biochemical in *Flavi* compared to G1. This is evidenced by the fact that the addition of B1 and G1 to the medium resulted in the breakdown of B1 for all types of isolates (except MKA01K), whereas G1 was not digested in most isolates (Figure 2D–F). In this regard, overall, the extrinsic introduction of B1 and G1 resulted in higher maximum levels of G1 for low and high producers in contrast to B1, whose average or maximally produced levels by fungi were reduced. The proposed preference of B1 to G1 in the antioxidative AF modulation in the *Flavi* environment may also be indicative of why B1 is usually found in a significant proportion of total AF in a number of *Flavi* strains [51,52,53,54,55] (Supplementary Table S1). B1 is also seen to be produced in higher amounts in a good number of strains studied compared to the rest of the AF types [52,53]. The modulation of AF levels can also be noted from the previous investigation in which *A. parasiticus* degraded AF through peroxidase enzyme [20] despite being a producer of the AF.

As regards the degradation of AF by non-producer strains, in their previous study, [33] showed that atoxigenic strains of *Flavi* digest B1. In our study, we furthermore show that low producers of B1 similarly do digest B1. Furthermore, we demonstrate that the mechanism of this digestion may not be the same between atoxigenic and toxigenic *Flavi*. While the low and high producers degraded the B1 in an antioxidative mechanism, the non-producers did not seem to degrade it in such a mechanism. This is explained by the fact that a significant increase in B1 is observed for low and high B1 producers when antioxidant (Se) is introduced to the environment alongside extrinsic B1. This is in comparison to the introduction of extrinsic B1 without Se. The increase is, however, not observed for non-producers between the two treatments (“Spp+Afl” v “Spp+Afl+AOx”, *p* > 0.05; Table 4). On the contrary, Se had no effect on the digestion of the B1 in non-producers, which sustained its digestion to similar levels as without the addition of Se (Figure 2A).

From a practical perspective, our findings also suggest the presence of antioxidants in the kernel before *Flavi* infection may deter an increase in AF levels in the kernel above certain limits. This is in comparison to the introduction of the antioxidant when AF and toxigenic *Flavi* are already present in the kernel (model “Spp”:“Spp+AOx”). This would imply that alteration of the abiotic environment of the kernel at preharvest by the introduction of non-toxic levels of mineral Se-antioxidant may be useful in deterring spikes in AF contamination. The approach would be problematic if the introduction of Se is at postharvest, such that a product that already has AF is biofortified with Se and gets contaminated with toxigenic *Flavi*. This would equate to the model “Spp”:“Spp+Afl+AOx” in our investigation, potentially leading to a spike in B1 and G1. Furthermore, our findings may also partly explain why a study on Brazilian nuts showed a higher accumulation of AF in nuts with higher levels of Se than those with lower levels of the antioxidant [56]. Considering that nuts are soil-borne, they are likely to get contaminated with aflatoxigenic *Flavi* and, subsequently, AF in the early stages of seed growth. This then renders the AF produced by *Flavi* unutilised in an antioxidative role due to the Se in the seed. This resonates with the observed higher levels of B1 and G1 in our study under the scenario “Spp+Afl+AOx.”

It should be noted that for the complete impediment of *Flavi*, higher levels of the antioxidant Se would have to be administered such that the *Flavi* fitness is reduced through non-sporulation [57,58] and subsequently prevent the AF production by the *Flavi*. The increase in the antioxidative capacity of a plant has been suggested previously as a means of reducing the oxidative stress in *Flavi* and, subsequently, AF production [22]. The antioxidative capacity of a plant by using elements such as Se would have to be high enough. This is as shown by our findings that the non-lethal dose (0.86 µg/g) did not deter AF production (on YES medium) in *Flavi*, although it reduced the maximum B1 levels produced. Higher Se antioxidative capacity can be achieved through the use of the nanoparticle format of Se. Se nanoparticles in higher levels are less toxic to organisms than mineral Se [59].

#### Gene Expression in High Aflatoxin B1/G1 Producer Isolates in Response to Antioxidant (Se)

From the results of two randomly selected high-producer strains (soil isolate ELG33C; maize isolate MLV14F), we demonstrated that the two biosynthetic genes *aflR* (regulatory) and *aflD* (structural) were consistently either both upregulated or downregulated in response to the abiotic stimulus Se-antioxidant. Furthermore, we observed a positive correlation in isolate ELG33C between expression levels of both *aflD* and *aflR* and the levels of B1 produced. The consistency was, however, not observed in isolate MLV14F as well as the G1 levels for both isolates (Figure 3).

Although the transcription factor *aflR* and structural gene *aflD* are involved in AF production in *Flavi*, most studies have shown no correlations between the gene expression levels in the two genes with levels of AF produced [60,61,62,63,64]. While some few studies have shown a level of correlation with AF levels produced, the studies have either been inconsistent with each other or the expression inconsistent with the expected highest production conditions for AF [65,66,67]; for example, high expression of *aflD* at both lowest B1 levels and lowest *aflR* expression and vice versa [65]. This may show that response to stimuli may be strain dependent, for example, the observed strain differences in response to CO_2_ [68] or temperature [64]. The possible strain dependence is also seen in our study on expression patterns of *aflD*/*aflR* to Se stimulus (Figure 3). In our study, the levels of available mRNA from *aflD* and *aflR* differed from the specific induced levels of Se as an antioxidant. Our preliminary findings show that the soil isolate ELG33C downregulated the two pathway genes at elevated control concentration (5 µg/g) after the preliminary upregulation at 0.40 µg/g. On the other hand, the maize isolate MLV14F only upregulated the two pathway genes at an elevated control concentration of 5 µg/g with no significant change at 0.40 µg/g. This would suggest that the downregulation of the genes in isolate MLV14F may probably occur at a higher Se level than the control-elevated concentration of 5 µg/g. A higher isolate sample size in our study would be required in order to fathom the pattern of gene expression–aflatoxin production correlation with antioxidants. An additional factor most investigators suggest in order to improve the correlation between gene expression and abiotic stimulus is the prevention of oxidative elements that could degrade the AFs in the medium in the course of the experiment, leading to poor or inconsistent correlations.

### 4.2. Fitness Response of Atoxigenic and Toxigenic Isolates under Antioxidant (Se) Treatment

At the maximum non-lethal dose of mineral Se administered (0.86 µg/g), we demonstrate the increase in fitness of the tested atoxigenic strains of *Flavi* compared to toxigenic counterparts, rejecting our initial hypothesis. Different fungi can thrive differently under the same abiotic environment or, indeed, under different abiotic environments. As seen in this investigation, introduction of non-lethal dose Se triggered an increase in spore production in the *Flavi*. We may attribute the relatively higher Darwinian fitness in atoxigenic than toxigenic *Flavi* to the presumption that the toxigenic *Flavi* may have channeled the Se to both sporulation and oxidative stress alleviation, whereas the atoxigenic *Flavi* may have solely channeled the Se towards sporulation. Considering the alteration of the abiotic environment for *Flavi* in our study, such a comparative increase in fitness of atoxigenic compared to toxigenic counterparts could be an important attribute in promoting the population of toxigenic over atoxigenic strains. This provides the need to further investigate the inhibitory concentration of Se nanoparticles that would trigger a growth inhibition of toxigenic strains, while at the same time allowing some atoxigenic counterparts to grow without complete inhibition. This is considering that biocontrol of AF premised on competitive exclusion relies on atoxigenic *Flavi* to outgrow toxigenic counterparts and colonise host crops [69,70]. From a practical perspective, integrating pre-harvest Se-biofortification of maize can have prospects to play a useful role as part of a broader AF biocontrol strategy.

## 5. Conclusions

While atoxigenic *Flavi* are known to degrade B1 in their environment, as seen in this study, toxigenic *Flavi* of this investigation modulated B1 in an antioxidative role by doing so to levels less than what they produced. We furthermore see that B1 happened to be a preferred antioxidative biochemical for the *Flavi* compared to G1. Furthermore, we do observe that atoxigenic *Flavi* used in this study were fitter than toxigenic counterparts under antioxidant treatment, contrary to expectations but a positive attribute. Hence, work may need to be performed on the prospective use of antioxidative elements, such as selenium (Se) in nanoparticle form, to drive a better fitness of atoxigenic fungi over toxigenic strains. This would be in the form of a broader biocontrol strategy in the competitive exclusion of *Flavi*. Furthermore, it should also be noted that when considering the use of antioxidants such as Se in substrates such as maize, consideration should be made to the AF contamination status of the substrate. From a practical perspective, Se biofortification may preferably be performed at preharvest on maize, or at postharvest when the product is without *Flavi* and AF contamination. This is because, while Se could deter potential spikes in B1 and G1 as observed in this study, its application to an environment already harbouring B1 and *Flavi* can conversely lead to a spike in the dreaded B1 when conditions are conducive for *Flavi* to proliferate.

## Figures and Tables

**Table 1:** *Flavi* isolates used in determination of B1 and G1 degradation.

Isolate	Aflatoxin-Production	Mean Aflatoxin Produced, ng/mL	
		B1	G1
1MS7 ^a^	None	0	0
125GF8 ^a^	None	0	0
ESF24B	None	0	0
MLV12B	None	0	0
ESF62A ^b^	Low	0	0
ELV13C	Low	3.2	8.0
MKZ06B	Low	33.8	0
EKZ10A	Low	52.1	146.3
MKA01K	High	342.4	83.9
EKW36B	High	164.6	323.3
EKW40A	High	1340.2	527.6
ELG33C	High	6749.8	10.4

^a^ Obtained as lab culture reference isolates from other experiments. ^b^ Was assigned as low producer, considering that AF was produced in presence of antioxidant. Strain coding first letter is ‘E’ for soil and ‘M’ for maize. The aflatoxins B1 and G1 were determined by High-Pressure Liquid Chromatography.

**Table 2:** Primers used in quantitative PCR assays for determination of gene expression in *aflD* and *aflR*.

Gene	Primer Name	Primer Pair Nucleotide Sequence	Position *	GenBank Accession No.	Reference
*aflR*	*AflR* taq1	(F)—TCG TCC TTA TCG TTC TCA AGG	1646	AF441435.2	[46]
	*AflR* taq2	(R)—ACT GTT GCT ACA GCT GCC ACT	1735		
*aflD*	Nor taq1	(F)—GTC CAA GCA ACA GGC CAA GT	516	XM_002379908.1	[47]
	Nor taq2	(R)—TCG TGC ATG TTG GTG ATG GT	562		
β-tubulin	Ben taq 1	(F)—CTT GTT GAC CAG GTT GTC GAT	65	AF036803.1	[46]
	Ben taq 2	(R)—GTC GCA GCC CTC AGC CT	99		

* Positions are according to the published sequences of the above genes of *A. flavus*.

**Table 3:** Aflatoxin digestion in *Flavi* according to levels of B1 and G1, ng/mL (without antioxidant treatment).

Toxigenicity	AF Digestion: [A] “Afl” v “Spp+Afl”		[B] Effect on AF Max Levels If AF was Not Reduced in [A]: [“Spp” v “Spp+Afl”]	
	B1	G1	B1	G1
None	Reduced, *p* = 0.029	No reduction, *p* = 0.114	Not applicable	Not applicable
Low	Reduced, *p* = 0.029	No reduction, *p* ~ 0.99	Not applicable	Not applicable
High	No reduction, *p* = 0.34	No reduction, *p* = 0.34	Max produced reduced (33,749 to 3169)	Max produced exceeded (2638 to 4914)

**Table 4:** Aflatoxin digestion in *Flavi* according to levels of B1 and G1, ng/mL due to antioxidant treatment.

Aflatoxin-Production	AF Utilisation: [C] “Spp+Afl” v “Spp+Afl+AOx”		AF Suppression: [D] Effect on Se on Max Levels [“Spp” v “Spp+AOx”]	
	B1	G1	B1	G1
None	Se no effect, B1 reduction same *p* = 0.69	Se no effect, No G1 reduction same *p* = 0.69	Not applicable	Not applicable
Low	B1 utilisation reduced by Se, *p* = 0.03	G1 non-utilisation unaffected by Se, *p* = 0.11	No effect, *p* = 0.89; B1 Max produced not exceeded (260 to 214)	No effect, *p* = 0.66; G1 Max produced not exceeded (732 to 463)
High	B1 utilisation not affected by Se, *p* = 0.11	G1 non-utilisation unaffected by Se, *p* = 0.49	No effect, *p* = 0.89; B1 Max produced not exceeded (33,749 to 25,842)	No effect, *p* ~ 0.99; G1 Max produced is exceeded (2638 to 4612)

## Data Availability

Code for the aflatoxin degradation/modulation analyses is found at https://github.com/bkatati/afloxidate (accessed on 27 April 2023). The lab culture isolates used in the experiment were stored at −70 °C, available for re-use.

## Supplementary Materials

The following supporting information can be downloaded at https://www.mdpi.com/article/10.3390/jof9060690/s1. Table S1: Percentage (%) of aflatoxin-producing ability of isolates.

## References

[1] Ndisio B., Peter W., Victor K., Sheila O., Boaz N., Wachira P., Kagot V., Okoth S. (2017). Susceptibility of locally cultivated groundnut (*Arachis hypogaea*) varieties to aflatoxin accumulation in Homa Bay County, Kenya. Afr. J. Microbiol. Res..

[2] Mallikarjunaiah N.H., Jayapala N., Puttaswamy H., Ramachandrappa N.S. (2017). Characterization of non-aflatoxigenic strains of *Aspergillus flavus* as potential biocontrol agent for the management of aflatoxin contamination in groundnut. Microb. Pathog..

[3] Kachapulula P.W., Akello J., Bandyopadhyay R., Cotty P.J. (2017). Aspergillus section Flavi community structure in zambia influences aflatoxin contamination of maize and groundnut. Int. J. Food Microbiol..

[4] Munkvold G.P., Arias S., Taschl I., Gruber-Dorninger C. (2019). Chapter 9: Mycotoxins in corn: Occurrence, impacts, and management. Corn.

[5] Akello J., Ortega-Beltran A., Katati B., Atehnkeng J., Augusto J., Mwila C.M., Mahuku G., Chikoye D., Bandyopadhyay R. (2021). Prevalence of Aflatoxin- and Fumonisin-Producing Fungi Associated with Cereal Crops Grown in Zimbabwe and Their Associated Risks in a Climate Change Scenario. Foods.

[6] WHO (2018). Safety Evaluation of Certain Contaminants in Food: Prepared by the Eighty-Third Meeting of the Joint FAO/WHO Expert Committee on Food Additives (JECFA).

[7] IARC (2012). Agents Classified by the IARC Monographs; International Agency for Research on Cancer, vol. 1–104. https://monographs.iarc.who.int/list-of-classifications.

[8] Rushing B.R., Selim M.I. (2019). Aflatoxin B1: A review on metabolism, toxicity, occurrence in food, occupational exposure, and detoxification methods. Food Chem. Toxicol..

[9] Li C., Liu X., Wu J., Ji X., Xu Q. (2022). Research progress in toxicological effects and mechanism of aflatoxin B_1_ toxin. PeerJ.

[10] Mohsenzadeh M.S., Hedayati N., Riahi-Zanjani B., Karmi G. (2016). Immunosuppression following dietary aflatoxin B1 exposure: A review of the existing evidence. Toxin Rev..

[11] Zhao Y., Wang T., Li P., Chen J., Nepovimova E., Long M., Wu W., Kuca K. (2021). *Bacillus amyloliquefaciens* B10 can alleviate aflatoxin B1-induced kidney oxidative stress and apoptosis in mice. Ecotoxicol. Environ. Saf..

[12] Gallo A., Giuberti G., Frisvad J., Bertuzzi T., Nielsen K. (2015). Review on Mycotoxin Issues in Ruminants: Occurrence in Forages, Effects of Mycotoxin Ingestion on Health Status and Animal Performance and Practical Strategies to Counteract Their Negative Effects. Toxins.

[13] Abdel-Hamid A.A., Firgany A.E.-D.L. (2015). Vitamin E supplementation ameliorates aflatoxin B1-induced nephrotoxicity in rats. Acta Histochem..

[14] Rotimi O.A., Rotimi S.O., Duru C.U., Ebebeinwe O.J., Abiodun A.O., Oyeniyi B.O., Faduyile F.A. (2017). Acute aflatoxin B1—Induced hepatotoxicity alters gene expression and disrupts lipid and lipoprotein metabolism in rats. Toxicol. Rep..

[15] Wang Y., Liu F., Zhou X., Liu M., Zang H., Liu X., Shan A., Feng X. (2022). Alleviation of Oral Exposure to Aflatoxin B1-Induced Renal Dysfunction, Oxidative Stress, and Cell Apoptosis in Mice Kidney by Curcumin. Antioxidants.

[16] Engin A.B., Engin A. (2019). DNA damage checkpoint response to aflatoxin B1. Environ. Toxicol. Pharmacol..

[17] Drott M.T., Debenport T., Higgins S.A., Buckley D.H., Milgroom M.G. (2019). Fitness Cost of Aflatoxin Production in *Aspergillus flavus* When Competing with Soil Microbes Could Maintain Balancing Selection. mBio.

[18] Drott M.T., Lazzaro B., Brown D., Carbone I., Milgroom M. (2017). Balancing selection for aflatoxin in *Aspergillus flavus* is maintained through interference competition with, and fungivory by insects. Proc. Biol. Sci..

[19] Arai T., Ito T., Koyama Y. (1967). Antimicrobial Activity of Aflatoxins. J. Bacteriol..

[20] Doyle M.P., Marth E.H. (1979). Peroxidase activity in mycelia of Aspergillus parasiticus that degrade aflatoxin. Appl. Microbiol. Biotechnol..

[21] Fountain J.C., Bajaj P., Nayak S.N., Yang L., Pandey M., Kumar V., Jayale A.S., Chitikineni A., Lee R.D., Kemerait R.C. (2016). Responses of *Aspergillus flavus* to Oxidative Stress Are Related to Fungal Development Regulator, Antioxidant Enzyme, and Secondary Metabolite Biosynthetic Gene Expression. Front. Microbiol..

[22] Fountain J.C., Bajaj P., Pandey M., Nayak S.N., Yang L., Kumar V., Jayale A.S., Chitikineni A., Zhuang W., Scully B.T. (2016). Oxidative stress and carbon metabolism influence *Aspergillus flavus* transcriptome composition and secondary metabolite production. Sci. Rep..

[23] Jayashree T., Subramanyam C. (2000). Oxidative stress as a prerequisite for aflatoxin production by Aspergillus parasiticus. Free. Radic. Biol. Med..

[24] Narasaiah K.V., Sashidhar R.B., Subramanyam C. (2006). Biochemical analysis of oxidative stress in the production of aflatoxin and its precursor intermediates. Mycopathologia.

[25] Fountain J.C., Scully B.T., Ni X., Kemerait R.C., Lee R.D., Chen Z.-Y., Guo B. (2014). Environmental influences on maize-*Aspergillus flavus* interactions and aflatoxin production. Front. Microbiol..

[26] Rayman M.P. (2000). The importance of selenium to human health. Lancet.

[27] Kuršvietienė L., Mongirdienė A., Bernatonienė J., Šulinskienė J., Stanevičienė I. (2020). Selenium Anticancer Properties and Impact on Cellular Redox Status. Antioxidants.

[28] Alsuhaibani A.M.A. (2018). Functional role of selenium-fortified yogurt against aflatoxin-contaminated nuts in rats. Agric. Food Secur..

[29] Mughal M.J., Peng X., Kamboh A.A., Zhou Y., Fang J. (2017). Aflatoxin B1 Induced Systemic Toxicity in Poultry and Rescue Effects of Selenium and Zinc. Biol. Trace Element Res..

[30] Wang J., Lin L., Jiang Q., Huang W., Liu N. (2019). Effect of supplemental lactic acid bacteria on growth performance, glutathione turnover and aflatoxin B1 removal in lambs. Czech J. Anim. Sci..

[31] Kaur T., Vashisht A., Prakash N.T., Reddy M.S. (2022). Role of Selenium-Tolerant Fungi on Plant Growth Promotion and Selenium Accumulation of Maize Plants Grown in Seleniferous Soils. Water Air Soil Pollut..

[32] Naseem M., Anwar-Ul-Haq M., Wang X., Farooq N., Awais M., Sattar H., Malik H.A., Mustafa A., Ahmad J., El-Esawi M.A. (2021). Influence of Selenium on Growth, Physiology, and Antioxidant Responses in Maize Varies in a Dose-Dependent Manner. J. Food Qual..

[33] Maxwell L.A., Callicott K., Bandyopadhyay R., Mehl H., Orbach M., Cotty P. (2021). Degradation of aflatoxins B1 by atoxigenic *Aspergillus flavus* biocontrol agents. Plant Dis..

[34] Bock C.H., Cotty P.J. (1999). Wheat seed colonized with atoxigenic *Aspergillus flavus*: Characterization and production of a biopesticide for aflatoxin control. Biocontrol Sci. Technol..

[35] Medina A., Mohale S., Samsudin N.I.P., Rodriguez-Sixtos A., Rodriguez A., Magan N. (2017). Biocontrol of mycotoxins: Dynamics and mechanisms of action. Curr. Opin. Food Sci..

[36] Rao K.R., Vipin A., Venkateswaran G. (2020). Mechanism of inhibition of aflatoxin synthesis by non-aflatoxigenic strains of *Aspergillus flavus*. Microb. Pathog..

[37] Xing F., Wang L., Liu X., Selvaraj J.N., Wang Y., Zhao Y., Liu Y. (2017). Aflatoxin B 1 inhibition in *Aspergillus flavus* by *Aspergillus niger* through down-regulating expression of major biosynthetic genes and AFB 1 degradation by atoxigenic *A. flavus*. Int. J. Food Microbiol..

[38] Yu J., Bhatnagar D., Cleveland T.E. (2004). Completed sequence of aflatoxin pathway gene cluster in *Aspergillus parasiticus*. FEBS Lett..

[39] Yu J., Fedorova N.D., Montalbano B.G., Bhatnagar D., Cleveland T.E., Bennett J.W., Nierman W.C. (2011). Tight control of mycotoxin biosynthesis gene expression in *Aspergillus flavus* by temperature as revealed by RNA-Seq. FEMS Microbiol. Lett..

[40] Chang P.-K. (2004). Lack of interaction between AFLR and AFLJ contributes to nonaflatoxigenicity of *Aspergillus sojae*. J. Biotechnol..

[41] Price M.S., Conners S.B., Tachdjian S., Kelly R.M., Payne G.A. (2005). Aflatoxin conducive and non-conducive growth conditions reveal new gene associations with aflatoxin production. Fungal Genet. Biol..

[42] Woloshuk C.P., Foutz K.R., Brewer J.F., Bhatnagar D., Cleveland E.T., Payne A.G. (1994). Molecular characterization of aflR, a regulatory locus for aflatoxin biosynthesis. Appl. Environ. Microbiol..

[43] Georgianna D.R., Payne G.A. (2009). Genetic regulation of aflatoxin biosynthesis: From gene to genome. Fungal Genet. Biol..

[44] Ono E.Y.S., Da Silva M., Ribeiro R.M.R., Ono M.A., Hayashi L., Garcia G.T., Hirooka E.Y. (2010). Comparison of thin-layer chromatography, spectrofluorimetry and bright greenish-yellow fluorescence test for aflatoxin detection in corn. Braz. Arch. Biol. Technol..

[45] Abdollahi A., Buchanan R.L. (1981). Regulation of Aflatoxin Biosynthesis: Induction of Aflatoxin Production by Various Carbohydrates. J. Food Sci..

[46] Medina A., Rodríguez A., Magan N. (2015). Climate change and mycotoxigenic fungi: Impacts on mycotoxin production. Curr. Opin. Food Sci..

[47] Abdel-Hadi A., Carter D., Magan N. (2010). Temporal monitoring of the nor-1 (aflD) gene of *Aspergillus flavus* in relation to aflatoxin B1 production during storage of peanuts under different water activity levels. J. Appl. Microbiol..

[48] Livak K.J., Schmittgen T.D. (2001). Analysis of relative gene expression data using real-time quantitative PCR and the 2(-Delta Delta C(T)) Method. Methods.

[49] R Core Team (2013). R: A Language and Environment for Statistical Computing.

[50] Wickham H. (2016). Ggplot2: Elegant Graphics for Data Analysis.

[51] Donner M., Atehnkeng J., Sikora R.A., Bandyopadhyay R., Cotty P.J. (2009). Distribution of Aspergillus section Flavi in soils of maize fields in three agroecological zones of Nigeria. Soil Biol. Biochem..

[52] Kachapulula P., Akello J., Bandyopadhyay R., Cotty P. (2017). Aflatoxin contamination of groundnut and maize in Zambia: Observed and potential concentrations. J. Appl. Microbiol..

[53] Cotty P.J., Cardwell K.F. (1999). Divergence of West African and North American Communities of *Aspergillus* Section *Flavi*. Appl. Environ. Microbiol..

[54] Mohale S., Medina A., Rodríguez A., Sulyok M., Magan N. (2013). Mycotoxigenic fungi and mycotoxins associated with stored maize from different regions of Lesotho. Mycotoxin Res..

[55] Rocha L.d.O., Reis G.M., Braghini R., Kobashigawa E., de Araújo J., Corrêa B. (2012). Characterization of aflatoxigenic and non-aflatoxigenic strains of Aspergillus section Flavi isolated from corn grains of different geographic origins in Brazil. Eur. J. Plant Pathol..

[56] Pacheco A.M., Scussel V.M. (2007). Selenium and Aflatoxin Levels in Raw Brazil Nuts from the Amazon Basin. J. Agric. Food Chem..

[57] Asghari-Paskiabi F., Imani M., Rafii-Tabar H., Razzaghi-Abyaneh M. (2019). Physicochemical properties, antifungal activity and cytotoxicity of selenium sulfide nanoparticles green synthesized by *Saccharomyces cerevisiae*. Biochem. Biophys. Res. Commun..

[58] Hassan A.A., Iskander D., Oraby N.H. (2022). Evaluation of the synergistic antimicrobial activities of selenium nanoparticles and Rosemary oil against *Aspergillus fumigatu* and *Klebsiella pneumoniae* recovered from respiratory infection in cattle in Giza governorate, Egypt. Explor. Anim. Med Res..

[59] Bhattacharjee A., Basu A., Bhattacharya S. (2019). Selenium nanoparticles are less toxic than inorganic and organic selenium to mice in vivo. Nucleus.

[60] Accinelli C., Abbas H., Zablotowicz R., Wilkinson J. (2008). *Aspergillus flavus* aflatoxin occurrence and expression of aflatoxin biosynthesis genes in soil. Can. J. Microbiol..

[61] Al-Saad L.A., Al-Badran A.I., Al-Jumayli S.A., Magan N., Rodriguez A. (2016). Impact of bacterial biocontrol agents on aflatoxin biosynthetic genes, aflD and aflR expression, and phenotypic aflatoxin B(1) production by *Aspergillus flavus* under different environmental and nutritional regimes. Int. J. Food. Microbiol..

[62] Rodrigues P., Venâncio A., Kozakiewicz Z., Lima N. (2009). A polyphasic approach to the identification of aflatoxigenic and non-aflatoxigenic strains of Aspergillus Section Flavi isolated from Portuguese almonds. Int. J. Food Microbiol..

[63] Bernáldez V., Córdoba J.J., Magan N., Peromingo B., Rodriguez A. (2017). The influence of ecophysiological factors on growth, aflR gene expression and aflatoxin B 1 production by a type strain of *Aspergillus flavus*. LWT Food Sci. Technol..

[64] Yunes N.B.S., Oliveira R.C., Reis T.A., Baquião A.C., Rocha L.O., Correa B. (2020). Effect of temperature on growth, gene expression, and aflatoxin production by *Aspergillus nomius* isolated from Brazil nuts. Mycotoxin Res..

[65] Gallo A., Solfrizzo M., Epifani F., Panzarini G., Perrone G. (2016). Effect of temperature and water activity on gene expression and aflatoxin biosynthesis in *Aspergillus flavus* on almond medium. Int. J. Food Microbiol..

[66] Obrian G.R., Georgianna D.R., Wilkinson J.R., Yu J., Abbas H.K., Bhatnagar D., Cleveland T.E., Nierman W., Payne G.A. (2007). The effect of elevated temperature on gene transcription and aflatoxin biosynthesis. Mycologia.

[67] Chang P.-K., Wilkinson J.R., Horn B.W., Yu J., Bhatnagar D., Cleveland T.E. (2007). Genes differentially expressed by *Aspergillus flavus* strains after loss of aflatoxin production by serial transfers. Appl. Microbiol. Biotechnol..

[68] Baazeem A., Rodriguez A., Medina A., Magan N. (2021). Impacts of climate change Interacting abiotic factors on growth, aflD and aflR gene expression and Aflatoxin B1 production by *Aspergillus flavus* strains in vitro and on pistachio nuts. Toxins.

[69] Cotty P.J., Bhatnagar D. (1994). Variability among atoxigenic *Aspergillus flavus* strains in ability to prevent aflatoxin contamination and production of aflatoxin biosynthetic pathway enzymes. Appl. Environ. Microbiol..

[70] Zanon M.S.A., Clemente M.P., Chulze S.N. (2018). Characterization and competitive ability of non-aflatoxigenic *Aspergillus flavus* isolated from the maize agro-ecosystem in Argentina as potential aflatoxin biocontrol agents. Int. J. Food Microbiol..

