# Supplementary material for: Aflatoxigenic Aspergillus Modulates Aflatoxin-B1 Levels through an Antioxidative Mechanism
Source: J Fungi (Basel). 2023 Jun 20;9(6):690. doi: 10.3390/jof9060690 (PMC10302197; doi:10.3390/jof9060690)
Supplement: Supplementary file 1 [file jof-09-00690-s001.zip › jof-2397608-supplementary.pdf]

Supplemental Table S1. Percentage (%) aflatoxin producing ability of isolates

Isolate	Clade	Toxigenicity	G2	G1	B2	B1
EKW40A†	*parasiticus*	High	0.3	28.2	0.0	71.5
EKW36B	*parasiticus*	High	2.1	64.3	0.9	32.7
MKA01K†	*flavus*	High	0.6	19.6	0.0	79.9
ELG33C†	*parasiticus*	High	2.1	0.1	12.9	84.9
		**Mean**	**1.3**	**28.0**	**3.5**	**67.2**
EKZ10A	*parasiticus*	Low	2.2	72.2	0.0	25.7
MKZ06B†	*minisclerotigenes*	Low	0.0	0.0	0.0	100.0
ELV13C	*parasiticus*	Low	0.0	71.3	0.0	28.7
		**Mean**	**0.7**	**47.8**	**0.0**	**51.4**

† Depicts isolates that produced more B1 than G1. A significant proportion of total aflatoxin is B1 with 4 out of the 7 isolates producing B1 > 50%.
